# Comparison Between Fast-Track and Conventional Anesthesia for Children Undergoing Closure of Ventricular Septal Defects: A Systematic Review and Meta-Analysis

**DOI:** 10.7759/cureus.49171

**Published:** 2023-11-21

**Authors:** Mohamad Nour Nasif, Hidar Alibrahim, Noheir Ashraf Ibrahem Fathy Hassan, Sedra Dashan, Heba Haj Saleh, Yazan Khair Eldien Jabban, Rami Soliman, Waleed Farouk Mohamed, Ihab Gebaly Mohammed Gabr, Ahmed Bahaa Salem Ali Osman, Simon Nader, Reham AbuShady, Ashraf N.B. Boktor, Nivedita Nair, Marwa Mohamed Alhanafy, Asrar Rashid

**Affiliations:** 1 Laboratory Medicine, University of Aleppo, Faculty of Medicine, Aleppo, SYR; 2 Internal Medicine, University of Aleppo, Faculty of Medicine, Aleppo, SYR; 3 Internal Medicine, Aswan University, Faculty of Medicine, Aswan, EGY; 4 Hematology, University of Aleppo, Faculty of Medicine, Aleppo, SYR; 5 Medicine, University of Aleppo, Faculty of Medicine, Aleppo, SYR; 6 Internal Medicine, Damascus University, Faculty of Medicine, Damascus, SYR; 7 Pulmonology/Respiratory Medicine, National Institute of Chest and Allergy, Cairo, EGY; 8 Internal Medicine, Healthpoint Hospital, Abu Dhabi, ARE; 9 Critical Care, Burjeel Medical City, Abu Dhabi, ARE; 10 Emergency, Yasclinic Hospital, Abu Dhabi, ARE; 11 Urology and Andrology, New Medical Centre (NMC) Royal Hospital, Abu Dhabi, ARE; 12 Obstetrics and Gynecology, New Medical Centre (NMC) Royal Hospital, Abu Dhabi, ARE; 13 Family Medicine, LLH Hospital, Abu Dhabi, ARE; 14 Ophthalmology, New Medical Centre (NMC) Royal Hospital, Abu Dhabi, ARE; 15 Neuroscience, New Medical Centre (NMC) Royal Hospital, Abu Dhabi, ARE; 16 Pediatric Intensive Care Unit, New Medical Centre (NMC) Royal hospital, Abu Dhabi, ARE

**Keywords:** children, systematic review and meta-analysis, conventional anesthesia, fast-track cardiac anesthesia, supracristal ventricular septal defects

## Abstract

Ventricular septal defect (VSD) is common in pediatric patients. This study aimed to evaluate the safety and effectiveness of using fast-track anesthesia and comparing it to traditional anesthesia, among children undergoing a transthoracic device closure for VSD. A systematic review following the 2020 Preferred Reporting Items for Systematic Reviews and Meta-Analyses (PRISMA) guidelines was conducted. Relevant literature was identified through specific search terms in the Scopus, Medical Literature Analysis and Retrieval System Online (MEDLINE), Excerpta Medica database (Embase), Web of Science, The Cochrane Central Register of Controlled Trials (CENTRAL), and Google Scholar databases. The inclusion criteria focused on observational studies that compared fast-track anesthesia with conventional anesthesia in pediatric VSD closure cases. Data extraction, quality assessment, and meta-analysis were performed using standard differences in means.

Initially, 6,535 papers were identified, and subsequent screening of titles and abstracts led to the inclusion of four retrospective studies from a total of 51 studies. The analysis encompassed 477 patients, with 235 in the fast-track anesthesia group and 242 in the conventional anesthesia group. No statistically significant disparities were observed between the two groups concerning the operative duration and hemodynamic variations post-intubation or post-procedure (P >0.05). Nevertheless, the fast-track anesthesia group demonstrated significantly reduced healthcare expenses as well as shorter periods of mechanical ventilation, ICU stay, and overall hospitalization compared to conventional anesthesia (P <0.05). The use of fast-track anesthesia in combination with transthoracic device closure for VSD demonstrates a safe and effective approach for pediatric patients. This approach results in reduced healthcare costs (10,000 Renminbi (RMB)) and shorter durations of mechanical ventilation, ICU admission, and hospitalization compared to conventional anesthesia. Further clinical trials are necessary to confirm these results and assess long-term outcomes.

## Introduction and background

Ventricular septal defect (VSD) is the most common non-cyanotic congenital heart disease (CHD) among the pediatric population; it can be an isolated anomaly or a component of more complex cardiac disease (such as the tetralogy of Fallot) [1]. The inter-ventricular septum is divided into five parts: membranous, muscular, infundibular, atrioventricular, and inlet. Failure or errors in the development and fusion process in any of the mentioned parts during the development of the fetus’s heart lead to VSD in the malformed part. The membranous type is known to be the most common VSD type (80% of cases) [2]. Large VSD defects are often diagnosed in utero or early during the postnatal period. They manifest with signs and symptoms of congestive heart failure (CHF) and mainly necessitate prompt surgical intervention. On the other hand, small VSDs usually remain asymptomatic, and many of them disappear in the first year of life. However, they can also cause some serious complications, such as pulmonary hypertension, infective endocarditis, and aortic regurgitation [3].

Surgery is often required in infants who fail to gain weight, fail to thrive or develop CHF symptoms. This open-surgery procedure includes a sternotomy with cardiopulmonary bypass [4]. However, this procedure carried many dangerous complications, like large incisions, massive blood loss, and a longer in-hospital stay, which in turn, raised the need for less invasive procedures. Transthoracic device closure and transcatheter device closure, which are commonly done under the guidance of a transesophageal echocardiogram, have become common therapies to close VSDs, and great outcomes have been documented, particularly for membranous and muscular defects, as they exclude the need for cardiopulmonary bypass, limit the need for blood transfusion, and decrease the number of days required for recovery and hospitalization. Additionally, they involve only tiny incisions, which improve cosmetic outcomes and decrease rehabilitation time and post-procedural pain. Yet, despite this, it has been linked to several potential side effects due to foreign body implantation, including arrhythmias, vascular complications, and valvular injuries [5, 6].

Many previous studies have confirmed the non-inferiority of transthoracic device closure to conventional surgery [6-8], and they have indicated that it can be a safe and efficacious alternative to the traditional open surgery procedure.

The term ‘fast-track anesthesia’ refers to a type of anesthesia that aims to facilitate early tracheal extubation (typically within six hours after surgery), mitigate the length of the ICU and hospital stay, and reduce medical expenses and complications to achieve both patients' safety and quality improvement [9]. The goal of this type of anesthesia is to substitute long-acting opioids with short-acting opioids or to decrease the dose of long-acting opioids. These opioids, together with inhaled or IV short-acting anesthetics, accomplish a fast return to voluntary breathing and circulation [10]. Furthermore, the use of fast-track anesthesia in young children also decreases the need for inotropes and yields a better perioperative outcome [11].

Several studies have shown the practicability and safety of fast-track anesthesia for pediatric cardiac surgery; however, there is a dearth of evidence on the use of fast-track anesthesia for transthoracic device VSD closure [12]. Therefore, the focus of this study was on the safety and efficacy of using fast-track anesthesia among children going through transthoracic device closure of VSD and to compare these results with the usage of traditional anesthesia. The primary outcomes measured were changes in hemodynamics, hospital costs, duration of mechanical ventilation, ICU stay, and overall hospitalization.

## Review

Methods

Literature Search Strategy

This review and meta-analysis were conducted following the guidelines outlined in the Cochrane Handbook for Systematic Reviews of Interventions [13]. The findings were subsequently reported in accordance with Preferred Reporting Items for Systematic Reviews and Meta-Analyses (PRISMA) guidelines and registered under the International Prospective Register of Systematic Reviews (PROSPERO) with the registration number CRD42023405051. A digital search was done in the Medical Literature Analysis and Retrieval System Online (MEDLINE), Excerpta Medica database (Embase), Medline, Scopus, Web of Science, Google Scholar, and The Cochrane Controlled Register of Trials (CENTRAL) for studies published between January 1990 and March 2023. Databases were searched without regard to language using pertinent medical subject headings, free-text terms, and research type filters as needed. Key journals' advance-access articles were examined for relevant papers. To locate any more randomized controlled trials (RCTs), the reference lists of every review or each RCT were manually searched. Duplicates resulting from a conference abstract and a full-text paper that followed were disregarded.

The relevant search string was formulated for each bibliometric database used, and the general search string agreed upon is as follows: ((Fast-Track) OR (Fast Track) OR (Rapid Cardiac) OR (low dose opioid) OR (low-dose opioid) OR (Fast-tracking) OR (Fast tracking) OR (Ventilation) OR (Extubations) OR (Extubations) OR (ICU) OR (CCU) OR (Care Unit) OR (care units) AND (Ventricular Septal Defects) OR (VSD) OR (Ventricular Septal Defect) OR (Intraventricular Septal Defects) OR (Intraventricular Septal Defect))

Inclusion and Exclusion Criteria

Only original articles, such as observational studies and experimental studies, were included in this review. For a study to be included, it had to compare fast-track anesthesia against conventional modes of anesthesia during real-time operational procedures. There were no linguistic limitations. Studies from January 1990 through March 2023 have been included. Excluded studies were those for which complete data could not be obtained. This systematic review sought to answer the following question: “Is there any difference between fast-track anesthesia and conventional anesthesia in children undergoing closure of ventricular septal defects?" We only included studies that compared the outcomes between children who underwent closure of a VSD using fast-track anesthesia and those who underwent that surgery using conventional anesthesia.

In terms of outcomes, the primary focus will be on central venous pressure (CVP), mean arterial pressure (MAP), and heart rate (HR). These measurements will be assessed after intubation, during the skin phase, and post-procedure. Secondary outcomes will encompass operative time, mechanical ventilation duration, ICU stay, and overall hospitalization duration.

Case reports, case series, reviews, editorials, animal studies, adult studies, and non-English studies were excluded.

Selection of Articles and Data Extraction

Two independent reviewers (SD and HHS) thoroughly examined the article list from each database based on the title and abstract before reviewing the complete text in accordance with the aforementioned inclusion and exclusion criteria. EndNote reference management program, version 20.2.1 (Clarivate Analytics, London, UK), was used to eliminate duplicate articles.

In the chosen publications, any disagreements were settled by a third investigator (MNN). All publications, published and unpublished, were examined using Clinicaltrials.gov; however, only trials that had been published were taken into account in this analysis.

Data extraction for each study was carried out independently by two reviewers under the direction of two authors. Under the initial author's guidance, conflicts were resolved. The following baseline data were extracted: study ID, including the first author of each study and year of publication; number of patients; age of patients; body weight of patients; ventricular septal defect size; and occluder size.

The following data were extracted regarding trial characteristics: any RCTs or observational studies such as cross-sectional studies, case-control studies, and prospective and retrospective cohorts are included in this review. Participants included children undergoing closure for a VSD. Interventions were fast-track anesthesia, and controls were those who had conventional anesthesia. Regarding outcomes, the primary outcomes were CVP, MAP, and HR after intubation, during skin incision, and after the procedure, while the secondary outcomes were operative time, mechanical ventilation time, ICU time, and hospital stay.

Data Collection, Analysis, and Risk of Bias

A first electronic search was carried out using the search terms and data sets described above. Each trial (RCT) that was shortlisted had its complete text reviewed and evaluated by two reviewers, with any discrepancies being resolved by the other reviewer. To identify additional RCTs, we manually searched the references of all full-text papers, including those that were included and those that were excluded, as well as other recently published systematic reviews. Information regarding the listed studies was obtained to create the data extraction form. Since all the studies included in our analysis were cohort studies, we assessed the risk of bias (RoB) using the Newcastle-Ottawa Scale (NOS), which assigns a maximum score of nine points. The NOS scale comprises three key aspects: selection, comparability, and outcomes. The selection category scrutinizes four elements: representativeness of the exposed cohort, selection of the nonexposed cohort, ascertainment of exposure, and demonstration that the outcome of interest was absent at the beginning of the study. The comparability of cohorts is determined by the study's design or analysis, while the outcome category evaluates the assessment of outcomes, the duration of follow-up to capture outcomes, and the adequacy of cohort follow-up.

The NOS categorizes the study quality according to scoring (good: 7-9; moderate: 4-6; low: 0-3).

Statistical Analysis and Quality Assessment

Statistical analysis of pooled data was performed using Review Manager, version 5.4.1 (The Nordic Cochrane Centre, The Cochrane Collaboration, 2014, Copenhagen, Denmark). The dichotomous data extracted were analyzed using the Mantel-Haenszel method, while generic inverse variance was used for continuous data. Both the random and fixed effect models were tested; however, only the random effects model was implemented. A Higgins I2 statistic test was performed to assess heterogeneity, whereby a value greater than 75% was considered high heterogeneity. In cases of significant heterogeneity, a leave-one-out sensitivity analysis was conducted by removing one study at a time to see if any single study affected the results. An inverted funnel plot was constructed using the fixed effects model to visually analyze for any potential publication bias via the symmetry of data points. A two-sided P-value ≤0.05 was considered statistically significant in all cases.

Results

Study Selection

**Figure 1:**
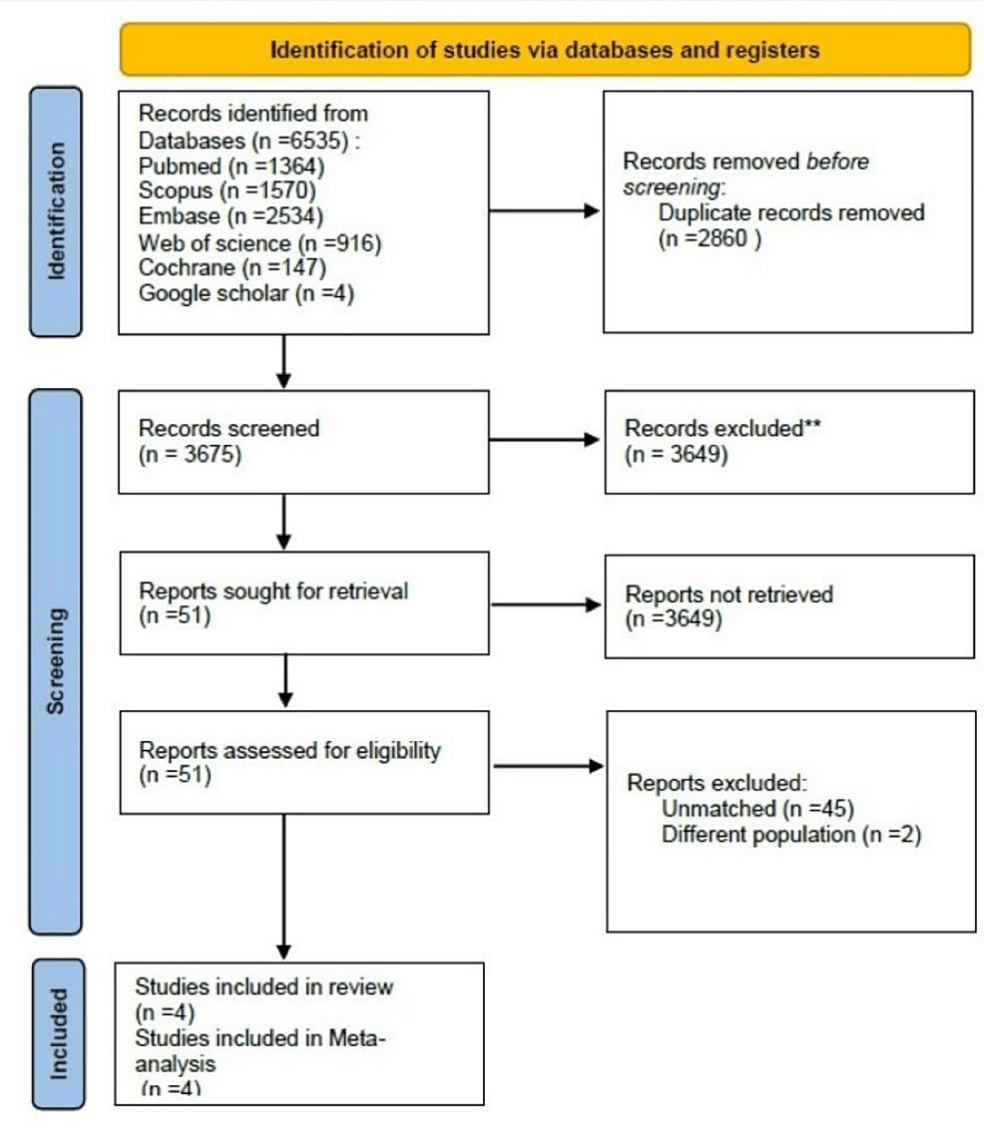
Flow chart of study selection based on PRISMA guidelines PRISMA: Preferred Reporting Items for Systematic Reviews and Meta-Analyses; Embase: Excerpta Medica database

Out of 6,535 papers, 2,860 papers were eliminated due to duplication. Then, 3,649 studies were excluded as they did not meet the inclusion criteria. Out of 51 studies, 45 were unmatched, and two other studies had different populations, so they were excluded; finally, four retrospective clinical studies were included in our meta-analysis (Figure 1).

Baseline Characteristics 

The patients were separated into two groups: Group F (rapid anesthesia) and Group C (conventional/standard anesthesia). A total of 477 patients were enrolled in the trial, with 235 in Group F and 242 in Group C. The baseline characteristics, such as age, gender, and body weight of patients per group in each study, are listed in Table 1, and we determined VSD size and occluder size.

**Table 1:** Baseline demographic data of the included studies Group F: fast-track anesthesia group; Group C: conventional anesthesia group; VSD: ventricular septal defect; M: male; F: female

Study	Year	Type of study	Patient groups	No of patients, n	Sex, n (%)	Age, Mean (SD)	Body Weight (kg)	VSD size (mm)	Occluder size (mm)	Pulmonary arterial pressure (mmHg)	Ejection fraction (%)	Operation time (min)	Mechanical ventilation time (h)	Intensive care time (h)	Hospital stay (days)
Xu et al. [10]	2021	Retrospective	Group F	35	M: 17 F: 18	3.01 ± 1.30	14.02 ± 0.89	5.03 ± 1.22	6.07 ± 1.68	-	-	48.00 ± 5.17	1.62 ± 0.53	4.81 ± 1.60	2.07 ± 0.74
			Group C	27	M: 15 F: 12	3.05 ± 1.17	14.24 ± 0.80	5.27 ± 1.17	6.57 ± 1.36	-	-	47.70 ± 5.09	4.38 ± 1.32	11.53 ± 2.47	4.63 ± 2.03
Yu et al. [12]	2019	Retrospective	Group F	42	-	3.31 ± 1.52	13.53 ± 5.78	5.03 ± 0.98	6.54 ± 1.13	-	-	-	-	-	-
			Group C	40	-	3.12 ± 1.72	12.87 ± 5.29	5.23 ± 1.45	6.62 ± 1.20	-	-	-	-	-	-
Zeng-Chun Wang et al. [14]	2020	Retrospective	Group F	30	M: 16 F: 14	3.3 ± 1.5	17.1 ± 4.1	4.5 ± 1.6	5.8 ± 1.9	-	-	-	-	-	-
			Group C	35	M: 18 F: 17	4.0 ± 2.1	16.7 ± 5.4	4.8 ± 1.3	6.3 ± 2.6	-	-	-	-	-	-
Wang et al. [15]	2018	Retrospective	Group F	128	M: 68 F: 60	2.5 ± 0.8	18.3 ± 2.5	4.8 ± 1.8	6.1 ± 1.3	33.8 ± 5.4	62.5 ± 5.2	-	-	-	-
			Group C	140	M: 76 F: 64	2.3 ± 1.0	18.9 ± 3.9	5.0 ± 1.5	6.3 ± 1.6	36.6 ± 7.9	65.1 ± 4.6	-	-	-	-

Difference in Standardized Means (SMD) of CVP, MAP, and HR Between Group F and C After Intubation

**Figure 2:**
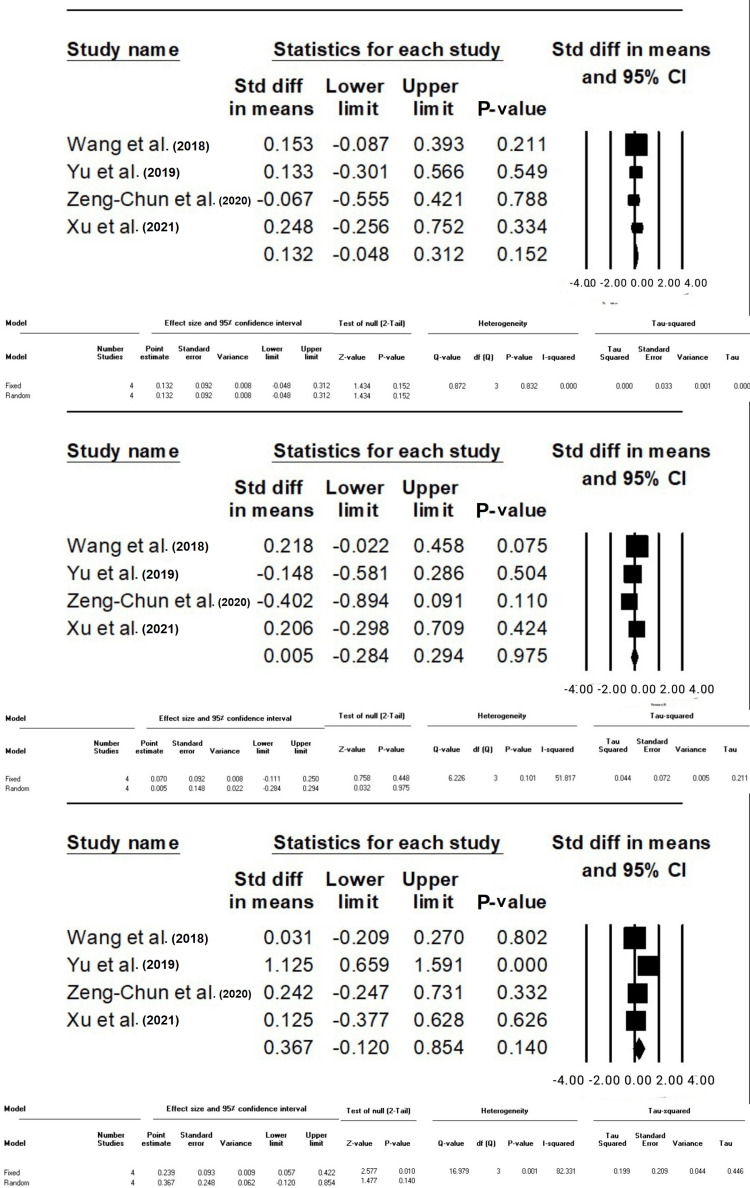
The standard difference in means between Groups F and C based on CVP, MAP, and HR after intubation, respectively. The data have been represented as mean±standard deviation with standard mean difference CVP: central venous pressure; MAP: mean arterial pressure; HR: heart rate Source: Xu et al. [10], Yu et al. [12], Zeng-Chun Wang et al. [14], Wang et al. [15].

The analysis revealed no statistically significant difference in CVP (SMD: 0.13, 95% CI: -0.04-0.31, P-value: 0.15), MAP (SMD: 0.005, 95% CI: -0.28-0.29, P-value: 0.9), and HR (SMD: 0.36, 95% CI: -0.12-0.85, P-value: 0.14) between Groups F and C following intubation (Figure 2).

Difference in SMD of CVP, MAP, and HR Between Group F and C After the Procedure

**Figure 3:**
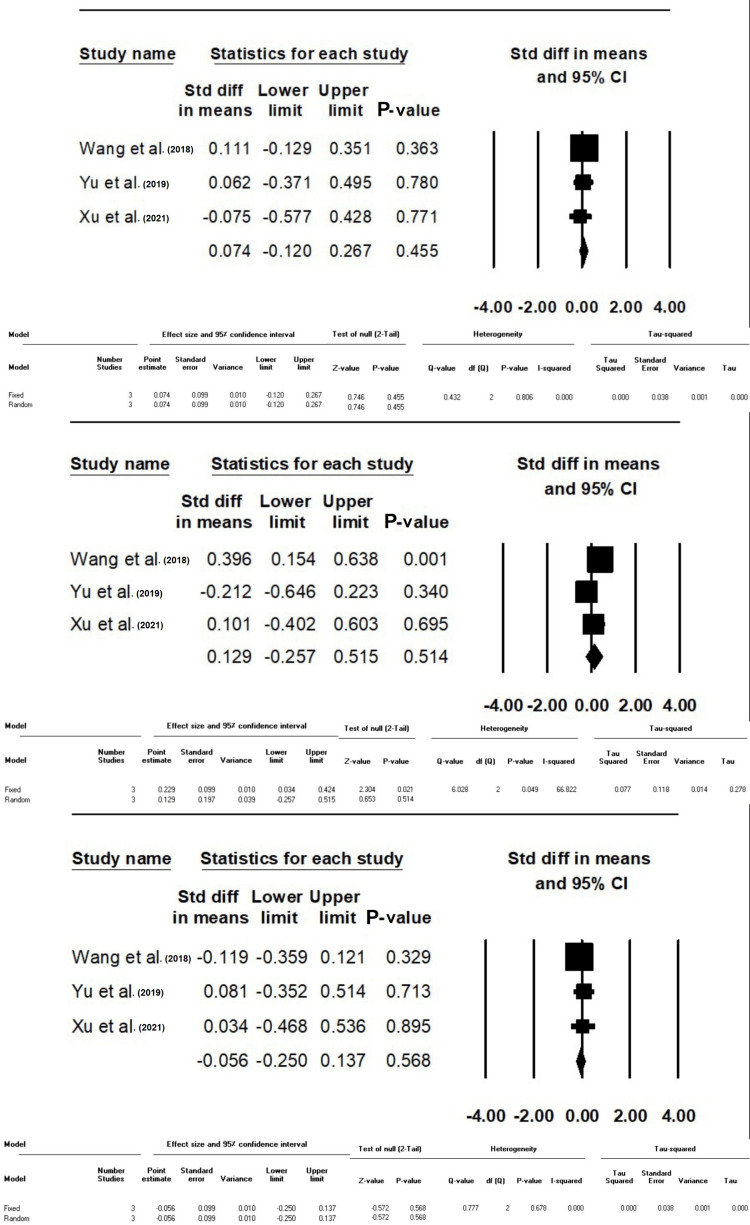
Standard difference in means between Groups F and C based on CVP, MAP, and HR after the procedure, respectively The data have been represented as mean±standard deviation with standard mean difference CVP: central venous pressure; MAP: mean arterial pressure; HR: heart rate Source: Xu et al. [10], Yu et al. [12], Zeng-Chun Wang et al. [14], Wang et al. [15].

This meta-analysis revealed no statistically significant difference in CVP (SMD: 0.07, 95% CI: -0.12-0.26, P-value: 0.45), MAP (SMD: 0.12, 95% CI: -0.25-0.51, P-value: 0.51), and HR (SMD: -0.05, 95% CI: -0.25-0.13, P-value: 0.56) between Groups F and C following the procedure (Figure 3).

The Difference in Means Between Groups F and C Based on Operative Time and Mechanical Ventilation Time

**Figure 4:**
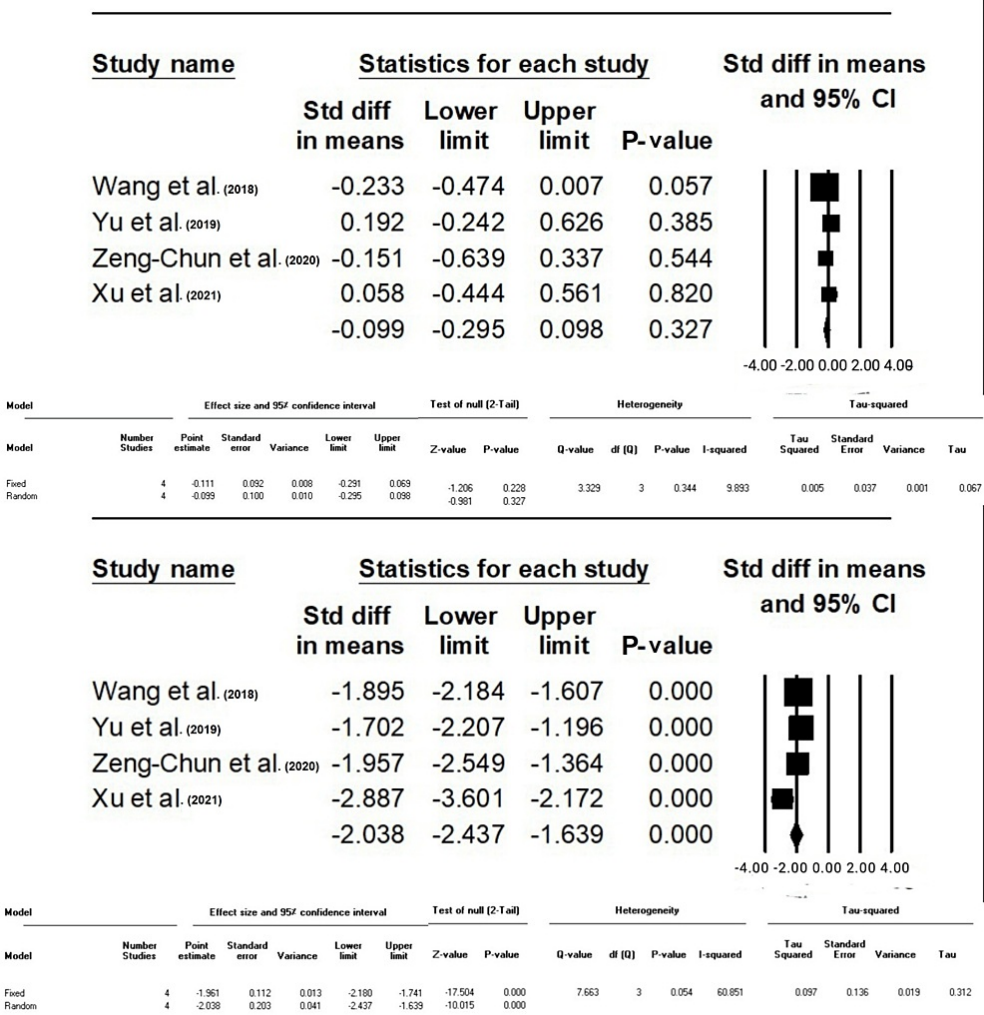
Standard difference in means between Groups F and C based on operative time (min) and mechanical ventilation time (hours), respectively. The data have been represented as mean±standard deviation with standard mean difference CVP: central venous pressure; MAP: mean arterial pressure; HR: heart rate Source: Xu et al. [10], Yu et al. [12], Zeng-Chun Wang et al. [13], Wang et al. [14]

Figure 4 showed no statistically significant difference in operative time between Groups F and C (p >0.05).

Nevertheless, in Group F, there was a statistically significant decrease in the duration of mechanical ventilation compared to Group C (SMD: -2.038, 95% CI: -2.4- (-1.6), P-value <0.001). Heterogeneity, as measured by I-squared (I^2^), was found to be moderate at 60.85% (P-value >0.05) (Figure 4).

The Difference in SMD of Hospital Stay Duration, ICU Stay Duration, and Medical Cost Between Groups F and C

**Figure 5:**
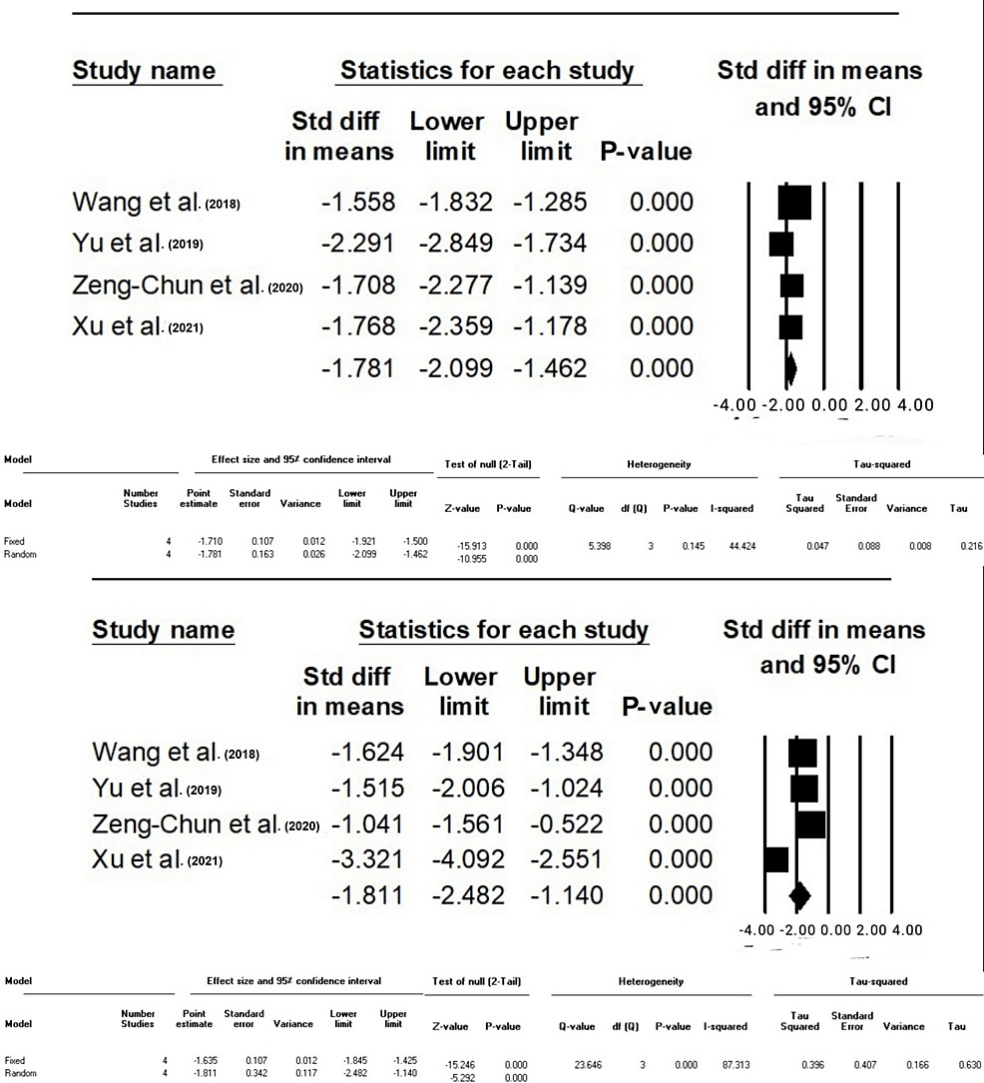
Standard difference in means between Groups F and C based on hospital stay time (d) and ICU stay time (h), respectively The data have been represented as mean±standard deviation with standard mean difference Source: Xu et al. [10], Yu et al. [12], Zeng-Chun Wang et al. [13], Wang et al. [14]

**Figure 6:**
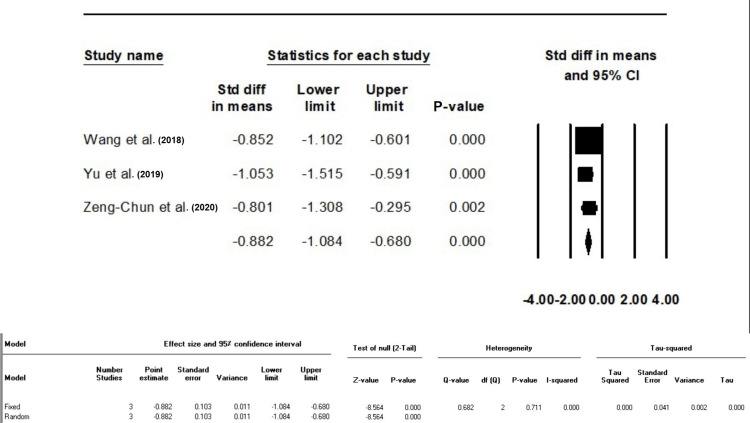
Standard difference in means between Groups F and C based on medical cost (10,000 RMB) The data have been represented as mean±standard deviation with standard mean difference RMB: Renminbi Source: Xu et al. [10], Yu et al. [12], Zeng-Chun Wang et al. [13], Wang et al. [14]

Group F showed a statistically significant decrease in both hospitalization (SMD: -0.17, 95%CI: -2 - (-1.4)) and ICU stay durations (SMD: -1.8, 95%CI: -2.4 - (-1.1)) compared to group C (p < 0.001); I^2^ was 40.42% for hospitalization time (P-value >0.05) and 87.31% for ICU stay time (P-value <0.001). In addition, Group F demonstrated a statistically significant reduction in medical costs compared to Group C (SMD: -0.88, 95%CI: -1.08 - (-0.6), P-value <0.001), with no heterogeneity (I^2^:0, P-value >0.05) (Figures 5, 6).

Quality Assessment of the Included Studies

Three studies were of good quality (score=7), and only one study was of moderate quality (score = 6) [1] (Table 2).

**Table 2:** Quality assessment of the included studies

New Castle-Ottawa scale assessment (NOS)		Selection				Comparability	Outcome			Quality score
Study ID	Name	Representativeness of the exposed cohort	Selection of the non-exposed cohort	Ascertainment of exposure	Demonstration that outcome of interest was not present at the start of the study	Comparability of cohorts on the basis of the design or analysis	Assessment of outcome	Was follow-up long enough for outcomes to occur	Adequacy of follow-up of cohorts	
1	Ning Xu MM et al.,2020 [10]		*	*	*	**	*			Moderate quality (6)
2	Ling-Shan Yu et al., 2019 [12]		*	*	*	**	*		*	Good quality (7)
3	Zeng-Chun Wang et al., 2020 [14]		*	*	*	**	*		*	Good quality (7)
4	Zeng-chunWang et al., 2018 [15]		*	*	*	**	*		*	Good quality (7)

Discussion

Ventricular septal defects are one of the most prevalent congenital cardiac abnormalities. Most cases of VSD are identified by pediatricians during regular checkups for heart murmurs soon after birth or in early childhood. The timing of VSD closure surgery and the kind of anesthesia used are determined by the defect's extent, the symptoms' existence, and the child's general health [12,15]. In this study, we aim to compare the efficacy and safety of fast-track anesthesia (Group F) versus conventional anesthesia (Group C) during VSD closure among children. The analysis indicated no difference in CVP, MAP, and HR after intubation and procedure, and no difference was detected in the operative time. However, fast-track anesthesia showed better results in reducing mechanical ventilation time, hospital stay time, ICU time, and medical costs than conventional anesthesia.

Consistent with our research, several studies have documented the advantages of fast-track anesthesia or enhanced recovery after surgery (ERAS) protocols for patients undergoing diverse surgical procedures [16-18]. These benefits include a shorter duration of hospitalization, earlier cessation of mechanical ventilation, expedited recovery, decreased healthcare expenditures, and decreased dependence on opioids for pain management.

Our findings are consistent with the findings of Meissner et al., who underlined the advantages of fast-track anesthesia in encouraging early and safe weaning from mechanical ventilation, hence reducing pulmonary issues among pediatric patients with congenital heart disease [19].

Moreover, Groesdonk et al.'s follow-up assessments at their heart center demonstrated the safety and reliability of fast-track anesthesia, as they observed no intraoperative consciousness when using ultra-short-acting opioids [20].

A study involving 613 young congenital heart disease patients found that 89% received fast-track anesthesia, which contributed to the expedited withdrawal from mechanical ventilation and shortened postoperative ICU and hospitalization stays [19].

The core principle of fast-track anesthesia entails minimizing opioid usage or combining short-acting opioids with other sedative analgesics or anesthetic techniques to achieve balanced anesthesia. The rapid-acting muscle relaxant, cisatracurium besylate, effectively inhibits histamine release, maintaining stable hemodynamics. Sevoflurane is colorless and mild. It is quickly eliminated and has low solubility in the blood. It is simple to use, prompts quick recovery, and protects cardiac tissue [21].

Remifentanil is an opioid receptor agonist with a short half-life. After ceasing the infusion, spontaneous breathing returns within three to five minutes due to its quick action. Additionally, it has a short half-life and is unaffected by alterations in infusion time or aging [12, 22]. Sufentanil, however, is a lipophilic opioid. It works quickly, has a potent analgesic effect, and has a half-life of around 150 minutes. The hemodynamics are only slightly impacted by it. The postoperative hemodynamic indicators in this investigation were steady in both groups, and no discernible difference was seen, supporting our findings [21].

The respiratory complications during extubation in the operating room and fast-track anesthesia compared to conventional anesthesia protocols for cardiac surgery are generally lower. Post-surgery practice reduces the necessity for muscle relaxants, promotes the restoration of spontaneous breathing, and mitigates the risks associated with iatrogenic lung inflammation, respiratory tract damage, and other pulmonary complications linked to mechanical ventilation [23]. A study utilizing propensity score matching has indicated that employing this approach, known as ultra-fast tract anesthesia (UFTA), in patients with low to moderate cardiac surgery risks results in enhanced cost-effectiveness and overall outcomes compared to traditional anesthesia methods [24]. Additionally, prospective observational research reveals that 87.1% of patients who underwent successful extubation in the operating room experienced no increased mortality or morbidity while benefiting from shorter stays in the ICU and reduced utilization of hospital resources [25].

For transthoracic VSD closure, fast-track cardiac anesthesia (FTCA) provides clear benefits over traditional anesthesia. A thorough assessment of their perioperative state should be performed to decide whether fast-track anesthesia is appropriate for pediatric patients [21].

Strengths and limitations

Almost all the studies included in our meta-analysis are of high quality, and no observable heterogeneity between the studies was found. However, there were a small number of studies with a small sample size, which is not representative of the whole community, and all these studies were retrospective. Adjusting for confounding variables was not done. More multicenter randomized studies are required to validate our results.

## Conclusions

Our comprehensive meta-analysis sheds light on the advantages of fast-track anesthesia in children undergoing closure of VSDs. Our findings reveal a significant reduction in medical expenses associated with the implementation of fast-track anesthesia, coupled with notable decreases in intensive care unit stays, overall hospitalization durations, and the duration of mechanical ventilation. These outcomes underscore the potential for fast-track anesthesia to not only contribute to cost savings but also enhance the overall efficiency of the perioperative care pathway for pediatric patients undergoing VSD closure. It is noteworthy that despite these positive effects on resource utilization and patient recovery, our analysis did not identify significant differences in hemodynamic changes between fast-track and conventional anesthesia during the VSD closure procedure.
